# Lamellar macular holes: evolving concepts and propensity for progression to full thickness macular hole

**DOI:** 10.1186/s40942-020-00252-x

**Published:** 2020-09-29

**Authors:** Salim Zafar Asaad

**Affiliations:** Department of Ophthalmology, Burjeel Speciality Hospital, Al Kuwaiti street, Al Fayha, Sharjah, United Arab Emirates

**Keywords:** Epiretinal proliferation, Lamellar macular hole, Full thickness macular hole, Epiretinal proliferation

## Abstract

Currently the term lamellar macular hole (LMH) alludes to a wide spectrum of macular conditions including distinct clinical entities with different pathomorphologies. Classifications into subtypes, tractional and degenerative or based on the associated preretinal tissue had been proposed. Recent insights suggest that only lesions with tissue loss should be considered ‘true’ LMH and not those morphological changes caused by tractional forces. Inclusion of lesions with foveoschisis with contractile epiretinal membrane (ERM) in earlier studies on LMHs has resulted in imprecise information about its clinical course. This review provides an overview of the evolving concepts of LMHs and analyses its natural history from study cases in previously published literature.

## Background

There is currently no consensus about what constitutes ‘lamellar macular hole’ (LMH) and its definition. The term alludes to a wide spectrum of macular pathomorphologies. Gass in 1975 first used the term to describe complication of cystoid macular edema after cataract extraction [1]. In 2006 Witkin et al. [2] proposed optical coherence tomography (OCT) criteria for diagnosis of LMHs that were adopted by the International Vitreomacular Traction Study Group [3], which included irregular foveal contour, defect in the inner fovea, intraretinal split and intact photoreceptors. But this definition does not address the associated preretinal tissue or status of ellipsoidal layer which influence clinical course & prognosis. Govetto et al. in 2016 described two subtypes of LMHs which are clinically distinct; the first ‘tractional’ characterised by schitic separation of the neurosensory retina and the second ‘degenerative’ characterised by intraretinal cavitation with ellipsoidal zone defect [4]. They observed that tractional LMHs are associated with tractional epiretinal membrane (ERM) and degenerative LMHs with nontractional epiretinal proliferation. Many other authors have also described two types of preretinal tissue associated with LMHs (Table 1). The first type referred to as tractional ERM [5] /normal ERM [6] /conventional ERM [7] /typical tractional ERM [8] /standard ERM [9] appears tomographically as an irregular hyperreflective layer attached intermittently to the underlying retina and associated with tractional signs such as retinal wrinkling, thickening & intraretinal cysts. The second type referred to as thick ERM [2] /dense ERM [5] /thicker ERM [6] /lamellar hole-associated epiretinal proliferation or LHEP [7] /atypical epiretinal tissue [8] appears tomographically as a thick homogenous material of medium reflectivity universally adherent to the underlying retina and exhibits no evidence of traction.

**Table 1:** Nomenclature used by various authors to describe the two types of preretinal tissue associated with LMHs

Author	Tomographic appearance	
	Irregular hyperreflective layer attached intermittently to retina and associated with tractional signs	Thick homogenous material of medium reflectivity universally adherent to retina, no traction
Witkins et al. [2]	ERM	Thick ERM
Parolini et al. [5]	Tractional ERM	Dense ERM
Bottoni et al. [6]	Normal ERM	Thicker ERM
Pang et al. [7]	Conventional ERM	Lamellar hole-associated epiretinal proliferation (LHEP)
Schumann et al. [8]	Typical tractional ERM	Atypical epiretinal tissue
Govetto et al. [4]	Tractional ERM	Nontractional epiretinal proliferation
dell’Omo et al. [9]	Standard ERM	LHEP

Recently Hubschman, Govetto and other members of an international panel of vitreoretinal experts proposed that only lesions with apparent tissue loss should be considered as LMHs and therefore presence of foveal cavity with undermined edges mandatory for its diagnosis along with irregular foveal contour and other signs of foveal tissue loss like thinning and pseudo-operculum [10]. Optional criteria suggested were epiretinal proliferation, ellipsoid line disruption and foveal bump. This definition is similar to Govetto et al.’s description in 2016 of degenerative LMH, one of their proposed subtypes of LMH. Their other proposed subtype tractional LMH does not involve any actual tissue loss, the pathomorphologic changes caused by traction and therefore the panel proposed to name it as ‘ERM foveoschisis’. Hubschman et al. also proposed that thick ERM/dense ERM/atypical epiretinal tissue/LHEP be named ‘epiretinal proliferation’ [10]. Although the term LHEP is widely used we agree that it is not precise as the material has been found in conditions other than LMHs in full thickness macular holes (FTMH)s and at extrafoveal sites following trauma iatrogenic and accidental [7]. For this article we have used the nomenclature proposed by Hubschman et al. but understand that concepts, definitions and terminology may evolve and change in future. The pathogenesis of LMHs is poorly understood and will not be delved into here.

## Natural history & historical review

ERM traction can cause foveoschisis and the appearance of an irregular foveal contour that can be confused with LMH [10]. The pathogenesis of is similar to that of macular pseudoholes where the morphologic changes are cause by tractional forces exerted by the overlying ERM [4]. ERM foveoschisis is associated with other tractional signs like retinal thickening, wrinkling and intraretinal cysts. Foveoschisis in association with contractile epiretinal membrane is frequently misdiagnosed as LMH.

ERM is seen more frequently than epiretinal proliferation and associated with a variety of clinical conditions. ERM appear as a whitish-gray translucent sheet on the retinal surface whereas epiretinal proliferation is typically not visible on ophthalmoscopy. On tomography ERM appear as highly reflective usually thin line whereas epiretinal proliferation is seen as usually thick homogenous material of medium reflectivity. On cursory examination the isoreflective epiretinal proliferation can be mistaken as part of the retina because of a thin hyperreflective line often covering it which is mistaken as ERM [2, 5, 10]. ERM is intermittently attached with frequent skip areas of contact with the underlying internal limiting membrane (ILM) and associated with other tractional signs such as retinal wrinkling, thickening and intraretinal cysts. In contrast epiretinal proliferation is universally adherent to the underlying retina and exhibits no evidence of traction. Epiretinal proliferation is only found in conditions with defects extending to the middle retinal layers and appear contiguous with them [7]. They have been seen associated with LMH, FTMH and at sites of trauma iatrogenic or accidental. In LMHs epiretinal proliferation is seen around/surrounding the edges of the defect and moulds with the inner retinal anatomy [7]. Both ERM and epiretinal proliferation can co-exist manifesting mixed features [4, 7, 9, 10, 12].

Many studies have suggested that LMHs usually remain stable over time, very few evolving into FTMH [2, 6, 7, 9]. We reviewed previously published literature on LMHs addressing natural history to compare their study cases to the current definition as proposed by Hubschman et al. [10]. Earlier studies mostly did not describe the morphology of LMH in their study cases but later reports have usually specified the subtype of LMH or associated preretinal tissue making it possible to estimate the clinical course of true LMHs. Witkin et al. [2] observed 19 eyes with LMH of which of which 6 had normal ERM and 11 had thickened ERM (likely similar to true LMH as defined by Hubschman et al.). One eye progressed to a FTMH but it is not specified what type of ERM it had. Bottoni et al. [6] followed 34 eyes with LMH with two different types of ERM, thicker (likely similar to true LMH) and normal (likely similar to ERM foveoschisis as defined by Hubschman et al.). Of the 10 thicker ERMs and 24 normal ERMs one of each type progressed to FTMH after 6 and 15 months follow-up respectively. Pang et al. [7] reviewed 2030 eyes of which LHEP (epiretinal proliferation) was found in 60 of 197 eyes with LMH and 8 of 99 eyes with FTMH. We think it’s possible that the eyes with FTMH with epiretinal proliferation could have progressed from LMH with epiretinal proliferation. Recently formed idiopathic FTMH would not be expected have any pre-macular tissue, though ERM or epiretinal proliferation may develop after some period of time. In another study Pang et al. [11] compared 62 eyes with LMH with LHEP (epiretinal proliferation) and 83 eyes with LMH with conventional ERM (likely similar to ERM foveoschisis). During the mean follow-up period of 26 months only one eye progressed to FTMH but it is not specified from which group; otherwise functionally & morphologically the LHEP group fared worse. Compera et al. [12] reported a case of LMH with LHEP (epiretinal proliferation) that progressed to FTMH. dell’Omo et al. [9] analysed 84 eyes with LMH, of which 43 had standard ERM alone (likely similar to ERM foveoschisis), 11 had LHEP alone (likely similar to true LMH) and 30 had both. In the follow-up period 3 eyes in the LHEP group progressed to FTMH.

We present this case in which bilateral LMH with epiretinal proliferation progressed to FTMH in both eyes.

### Case report

**Fig. 1:**
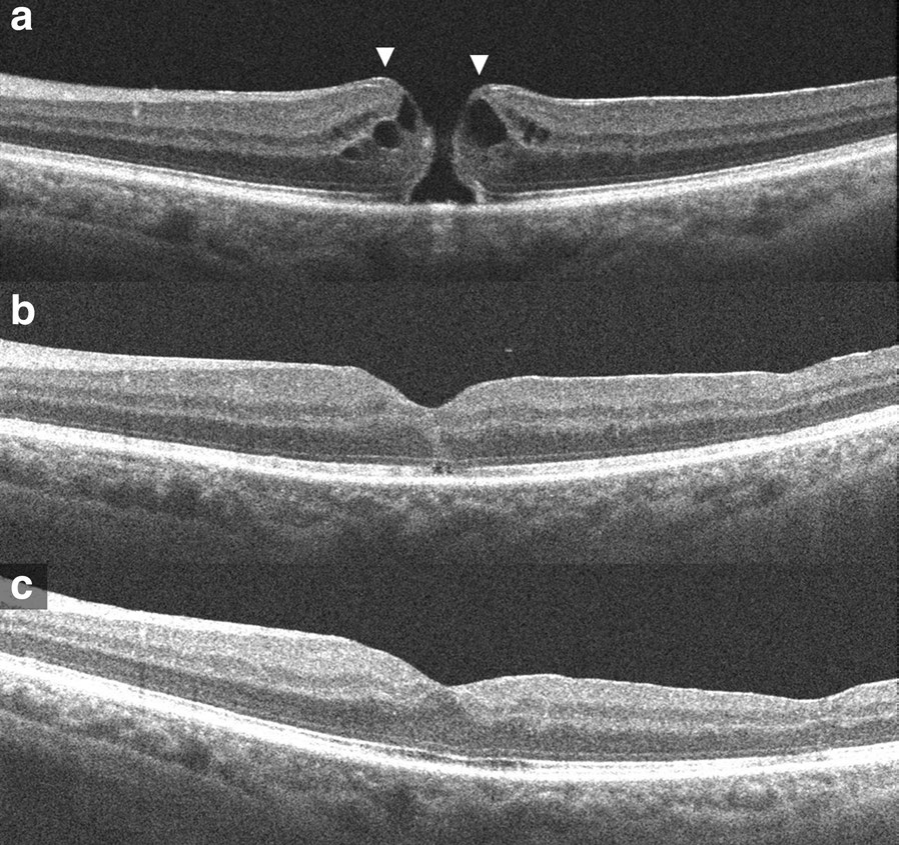
Optical coherence tomography images of the left eye, (**a**) On presentation; a full thickness macular hole with epiretinal proliferation at its edges seen as a homogeneous, isoreflective layer covered by a thin hyper-reflective line contiguous with inner retina (arrows). **b** At 1 month post-surgery; the macular hole is closed, a small outer retinal defect is seen. **c** At 2 months post-surgery; the outer retinal defect has resolved

**Fig. 2:**
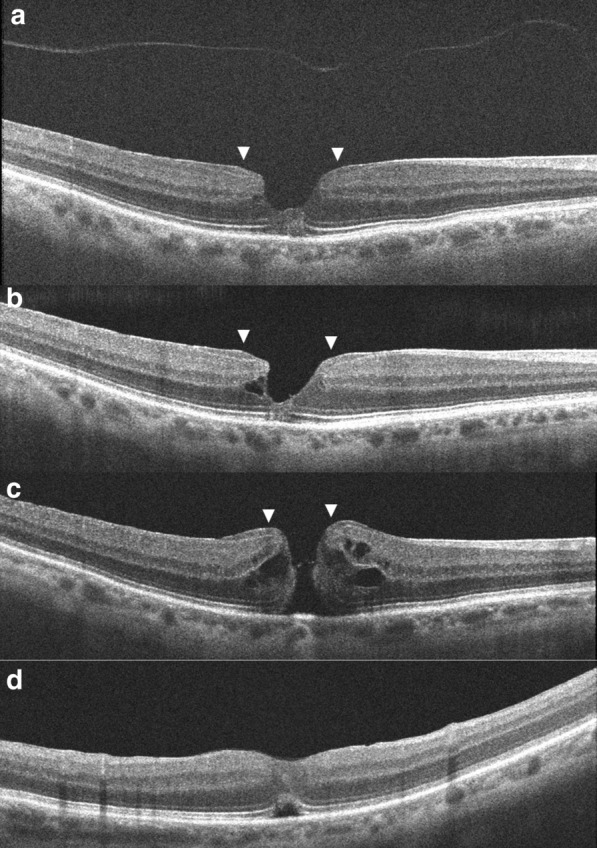
Optical coherence tomography images of the right eye, (**a**, **b**) On presentation; shows a typical lamellar macular hole with irregular foveal contour with thinning, foveal cavity, epiretinal proliferation, and disruption of ellipsoidal layer and external limiting membrane. Epiretinal proliferation is seen as a thick homogeneous, isoreflective layer covered by a thin hyper-reflective line at the edges of the hole contiguous with inner retina (arrows). The detached posterior hyaloid is also seen. **c** On occurrence of full thickness macular hole; epiretinal proliferation at the hole edges (arrows) appears contiguous with the inner retina (arrows). **d** At 1 month post-surgery; the macular hole is closed, an outer retinal defect is seen

A 72 year old male presented with increased blurring of vision in left eye since 1 week. He said he had blurred vision in both eyes since about a year. Best corrected visual acuity (BCVA) was 20/40 in right eye and 20/50 in left eye. Anterior examination showed pseudophakia both eyes, was otherwise unremarkable. Fundus examination showed FTMH in left eye, LMH in right eye with epimacular material in both eyes. OCT of left eye showed irregular margins of the macular hole with intraretinal edema. Epiretinal proliferation recognised as a homogeneous, isoreflective layer covered by a thin hyper-reflective line was seen at the edges of the hole contiguous with inner retina. Early ERM without tractional signs was also seen more centrifugally (Fig. 1a). OCT of right eye showed disruption of ellipsoidal layer and external limiting membrane and a cavitated appearance of the retina temporally. Epiretinal proliferation observed as a thick, homogeneous, isoreflective layer covered by a thin hyper-reflective line was seen at the surface of the hole edges and seemed contiguous with inner retina. ERM was seen centrifugally without tractional signs. The detached posterior hyaloid face was also visible in the scans (Fig. 2a, b). The patient underwent pars plana vitrectomy (PPV) with epiretinal proliferation and ERM peeling with ILM peeling with sulfur hexafluoride (SF6) in left eye. Successful closure of the macular hole was achieved. There was a small outer retinal defect (ORD) in the initial post-operative period which resolved spontaneously on follow-up (Fig. 1b, c). Visual acuity improved to 20/25 recorded 2 months post-operative. OCT of the right eye at this point showed further disorganization of central retina but visual acuity was maintained. He next presented again a month later with further deterioration vision in right eye since 1 day. BCVA was 20/60 in right eye and 20/25 in left eye. Examination showed a full thickness macular hole in right eye. OCT showed irregular edematous margins of the macular hole. Epiretinal proliferation seen at the hole edges appeared contiguous with the inner retina. ERM was also seen a bit centrifugally (Fig. 2c). He underwent PPV with epiretinal proliferation and ERM peeling with ILM peeling with SF6 in the right eye which achieved successful closure of the hole. An ORD and subretinal fluid (SRF) subfoveally was seen in the initial post-operative period but vision had improved to 20/32 (Fig. 2d). He was unable to follow-up after 1 month.

LMHs are invariably accompanied by ERM and/or epiretinal proliferation and can occasionally progress to FTMH [6, 7, 9, 11, 12, 15–18]. The pathogenesis of LMH progression to FTMH is poorly understood but it is evident that the vitreous plays no role. Conversely an idiopathic FTMH forms due to vitreofoveal traction with perifoveal vitreous detachment [13]. Thereafter ERM and/or epiretinal proliferation may develops around and have an adjunctive role in enlarging it [7, 14]. Thus when a FTMH is observed along with ERM/epiretinal proliferation and posterior vitreous detachment (PVD), it may not be obvious at first whether it occurred before the development of ERM/epiretinal proliferation or formed as a consequence of ERM/epiretinal proliferation with LMH. Tomographic images before the formation of FTMH if available would of course easily resolve this dilemma. Which in our case were available for the right eye documenting typical LMH before the occurrence of FTMH. Additionally the scans also show detached posterior hyaloid ruling out its role in subsequent FTMH formation. There are other signs which may indicate that a FTMH has evolved from LMH. The configuration of these holes might be suggestive of pre-existing inner retinal defect, distinct from that of idiopathic FTMH [15]. Finally the presence of epiretinal proliferation at the hole margins suggests it might have evolved from LMH as discussed earlier. In our case OCT scans demonstrate the presence of epiretinal proliferation in both eyes. The clinical presentation, sequential images and tomographic findings indicate that FTMH in both the eyes progressed from LMH with epiretinal proliferation.

## Surgical outcome

Surgery for LMHs has a variable outcome and generally not advocated. Earlier studies mostly did not describe the morphology of LMH in their study cases making it difficult to infer outcome of surgery in true LMH [2, 19–25] Later studies have usually specified the subtype of LMH or associated preretinal tissue making it possible to estimate outcomes in true LMHs. Parolini et al. followed 19 eyes that underwent vitrectomy with ERM and ILM peeling for LMH; 13 had dense ERM (epiretinal proliferation with likely true LMH) and 6 had tractional ERM (likely ERM foveoschisis). Surgery resulted in improvement of mean visual acuity in both groups but 3 eyes from the dense ERM group developed FTMH [5]. Lai et al. followed 43 cases of surgically treated LMH for over a year; 19 of them had LHEP (epiretinal proliferation). The pre and postoperative visual acuity showed no significant difference between the two groups [26]. Ko et al. reviewed 73 eyes that underwent vitrectomy for LMH. They reported no visual benefit in the 15 eyes which had presence of LHEP/epiretinal proliferation (likely true LMHs) [27]. dell’Omo et al. followed 26 eyes that underwent vitrectomy for LMH for a mean period of 32.8 ± 21.6 months; 14 had standard ERM alone, 4 had LHEP (epiretinal proliferation) alone and 8 had both. Postoperatively 3 eyes developed FTMH, 1 of them was from LHEP group. Final BCVA improved in all the groups and was not influenced by the presence of LHEP [9]. Coassin et al. followed 106 patients who underwent PPV with membranectomy and ILM peeling for LMH for a mean period of 36 months; 65% were tractional (likely ERM foveoschisis) and 35% degenerative (likely true LMH). Postoperatively visual acuity improved in the tractional group but not in the degenerative [28].

Surgery for FTMH with epiretinal proliferation has not been reported much in the literature. Pang et al. reported that in all the 3 cases of FTMH with LHEP (epiretinal proliferation) in their study group, there was successful macular hole closure after vitrectomy, ILM peel, intraocular tamponade and postoperative posturing. They observed that it is more difficult to start peeling LHEP than typical ERM describing it is more elastic and typically yellow [7]. Peeling of epiretinal proliferation might be not as important for relieving retinal traction as peeling ERM, but would have the function of allowing surgical access to the ILM [9]. Parolini et al. observed dense ERM (epiretinal proliferation) intraoperatively as having a yellow dense appearance and a fluffy consistency which could be completely separated from the retina and the ILM [5]. In our experience surgery for FTMH with epiretinal proliferation is not much different than that for FTMH with ERM. Except for the part about peeling the epiretinal proliferation which felt more friable and pliant, neither does it stain as well with Trypan blue. In our case, closure of FTMH with improvement in vision was achieved in both eyes after surgery despite ORDs in the initial post-operative period. The presence of central ORD with SRF on OCT after successful macular hole surgery is not uncommon, but does not impede visual improvement and usually resolves in few weeks [29, 30].

## Conclusion

Entities referred to as ‘tractional LMH’ and ‘LMH associated with tractional ERM’ should be excluded from the definition of LMHs as they are clinically, morphologically and pathogenically distinct. The key feature of LMHs is foveal tissue loss evidenced on OCT as foveal cavity with undermined edges and central foveal thinning. They are usually associated with epiretinal proliferation. ‘Mixed lesions’ have both LMH and ERM. Analysing previous reports by excluding tractional entities from their study population show that true LMHs have a worse functional, morphological outcome and may have a propensity to progress to FTMH. Surgery may not be an appropriate therapeutic strategy. Prospective studies on the natural history of LMHs and evaluating the role of epiretinal proliferation in the progression to FTMH are warranted to improve management of these lesions.

## Data Availability

The datasets used and/or analysed during the current study are available from the corresponding author on reasonable request.

## Abbreviations

FTMH: Full thickness macular hole
LMH: Lamellar macular hole
OCT: Optical coherence tomography
ERM: Epiretinal membrane
LHEP: Lamellar hole-associated epiretinal proliferation
PPV: Pars plana vitrectomy
ILM: Internal limiting membrane
SF6: Sulfur hexafluoride
ORD: Outer retinal defect
SRF: Subretinal fluid
PVD: Posterior vitreous detachment
BCVA: Best corrected visual acuity
